# HIV Care Providers’ Attitudes regarding Mobile Phone Applications and Web-Based Dashboards to support Patient Self-Management and Care Coordination: Results from a Qualitative Feasibility Study

**DOI:** 10.16966/2380-5536.127

**Published:** 2016-06-21

**Authors:** Dallas Swendeman, Shu Farmer, Deborah Mindry, Sung-Jae Lee, Melissa Medich

**Affiliations:** 1 Center for HIV Identification, Prevention, and Treatment Services (CHIPTS), Department of Psychiatry and Biobehavioral Science, David Geffen School of Medicine at UCLA, and Department of Epidemiology, UCLA Fielding School of Public Health, 10920 Wilshire Blvd., Suite 350, Los Angeles, CA 90024, USA; 2 Center for Culture and Health, Department of Psychiatry and Biobehavioral Science, NPI-Semel Institute for Neuroscience, 760 Westwood Plaza, Box 62, Los Angeles, CA 90024-1759, USA

**Keywords:** Mobile phones, Self-monitoring, Self-management, Care coordination, Healthcare providers, Psychosocial services providers, HIV care, Co-morbidities

## Abstract

In-depth qualitative interviews were conducted with healthcare providers (HCPs) from five HIV medical care coordination teams in a large Los Angeles County HIV clinic, including physicians, nurses, and psychosocial services providers. HCPs reported on the potential utility, acceptability, and barriers for patient self-monitoring and notifications via mobile phones, and web-based dashboards for HCPs. Potential benefits included: 1) enhancing patient engagement, motivation, adherence, and self-management; and 2) improving provider-patient relationships and HCP care coordination. Newly diagnosed and patients with co-morbidities were highest priorities for mobile application support. Facilitators included universal mobile phone ownership and use of smartphones or text messaging. Patient-level barriers included concerns about low motivation and financial instability for consistent use by some patients. Organizational barriers, cited primarily by physicians, included concerns about privacy protections, easy dashboard access, non-integrated electronic records, and competing burdens in limited appointment times. Psychosocial services providers were most supportive of the proposed mobile tools.

## Introduction

Mobile phones offer new opportunities for patients with chronic conditions to engage in self-management activities between clinical visits while also enabling real-time availability of data for health care providers (HCPs) to monitor patients’ status, provide more timely intervention, and coordinate care [1]. A small but growing number of studies examine health care providers’ (HCPs’) attitudes regarding mobile health (mHealth) adoption for patients’ remote monitoring and self-monitoring, and clinical dashboards using mHealth applications and data, particularly for patients with chronic illnesses [2–4]. Studies have examined HCPs’ attitudes regarding mobile phone applications for patient monitoring, self-management and clinical dashboards for asthma [5,6], diabetes [7], high blood pressure [8,9], and heart disease and failure [10–12]. These studies identify several perceived benefits by HCPs, including more timely data that is otherwise unavailable, the ability to respond more quickly than scheduled visits allow, and improved adherence and self-management by patients. These studies also identify potential barriers for HCPs such as time burdens, lack of reimbursements, lack of interoperability with other electronic systems, organizational or system readiness for adoption, and concerns about obligations (i.e., legal or liability risks) to respond immediately to patients’ crises or other data transmitted in real time to providers.

HCPs’ attitudes and support for technological innovations in health care are increasingly important given adoption of patient-centered medical home (PCMH) and care coordination models, in which care teams are responsible for managing patients’ comorbidities and related adherence and lifestyle behaviors. HIV care provides a case example with challenging co-morbidities and behavioral factors, and which is being integrated into primary health care in the United States, along with mental health, and drug abuse treatment, as supported by the Affordable Care Act [13–15]. Coinciding with the shift in medical homes and care coordination associated with the ACA, the Los Angeles County (LAC) Department of Public Health, Division of HIV and STD Programs, recently funded HIV Medical Care Coordination (MCC) programs throughout LAC. The MCC program supports multi-disciplinary care teams comprised of a physician or nurse practitioner (MD/NP), nurse case manager (NCM, registered nurse), licensed vocational nurse (LVN), and psychosocial case manager (PCMs, i.e., master’s level social workers or psychotherapists) to provide coordinated medical care and behavioral support in a medical home. The MCC program has an ultimate aim of supporting HIV-positive patients to become “self-managed” (i.e., achieve undetectable viral load and retention in care) by providing comprehensive and coordinated medical care and behavioral interventions for medication adherence, substance abuse, and mental health care [16,17].

Recent research has identified the potential efficacy of mobile phone self-monitoring and messaging for addressing common behavioral challenges and comorbidities for people living with HIV (PLH) to support self-management of mental health, reduction of substance use, decrease in sexual risk behaviors, and enhanced medication adherence [18,19]. Prior studies have examined HIV-patients preferences [20] and user-experiences [21] regarding mobile self-management applications, but research has not examined HIV care providers’ perspectives on feasibility, acceptability, preferences and barriers. The current study aims to begin to fill this gap in the research by examining HIV HCPs’ perspectives regarding the potential utility, acceptability, facilitators and barriers of mobile phone applications for patient self-monitoring and self-management support, and web-based clinical dashboards for provider support and care coordination with HIV-positive patients. More specifically, we examine HCPs’ perspectives from one of the largest HIV care and sexual health clinics in LAC, which was an early adopter of the MCC program one of the first sites open to considering adoption of mobile patient monitoring technologies.

## Methods

Study procedures were approved by the institutional review board (IRB) of University of California, Los Angeles. All members of all five MCC teams in a large HIV-treatment clinic embedded in a social services agency were contacted by the organization’s internal research staff to participate in 45- to 60-minute in-person interviews via announcements at in-service meetings, email, and outreach contacts. The stated purpose of the study was to examine the feasibility, acceptability, and suggestions for the development of mobile phone tools and clinical dashboards to support patient engagement in self-management for adherence, retention in care, and care coordination. Participants provided voluntary informed consent, agreed to have interviews audio-recorded, and were provided $30 remuneration for their time.

Participants included members of all five MCC teams, including four team leaders (three MDs, one Nurse Practitioner [NP]), two nurse case managers (NCMs, registered nurses) who focus on medication adherence and symptom management, two psychosocial case managers (PCMs) who provide brief behavioral interventions (i.e., for substance use, sexual risks, and referrals for mental health), and two licensed vocational nurses (LVNs) who conduct follow up contacts for adherence and retention. In addition, two staff ancillary to MCC teams also participated; one former PCM (LCSW) who was supervising retention in care program by outreach workers, and a master’s level psychotherapist coach for an intensive evidence-based behavioral intervention (EBI) for PLH disseminated by the CDC.

Interviews were guided by a semi-unstructured interview guide that first aimed to assess the roles and functions of the HCPs on the MCC teams. Then, barriers and facilitators to patient adherence, retention, and care coordination were discussed in order to inform feedback on hypothetical mobile phone self-monitoring and messaging tools, and web-based clinical dashboards for medication adherence, physical and mental health symptoms, substance use, and sexual risk behaviors. Interviews then specifically focused on discussion of HCPs’ general interest and perspectives on potential feasibility, acceptability, facilitators, barriers, and suggestions for application functions, priority domains, and priority patient sub-groups.

Interviews were transcribed and coded by the authors using a grounded theory approach, which involves working from the data to identify key themes or descriptive codes and subthemes within the data [22]. Coding was conducted using Dedoose online software, version 4.5.91 [23]. This paper focuses on results related specifically to hypothetical mobile applications and clinical dashboards, rather than patient adherence and retention more generally.

## Results

**Figure 1:**
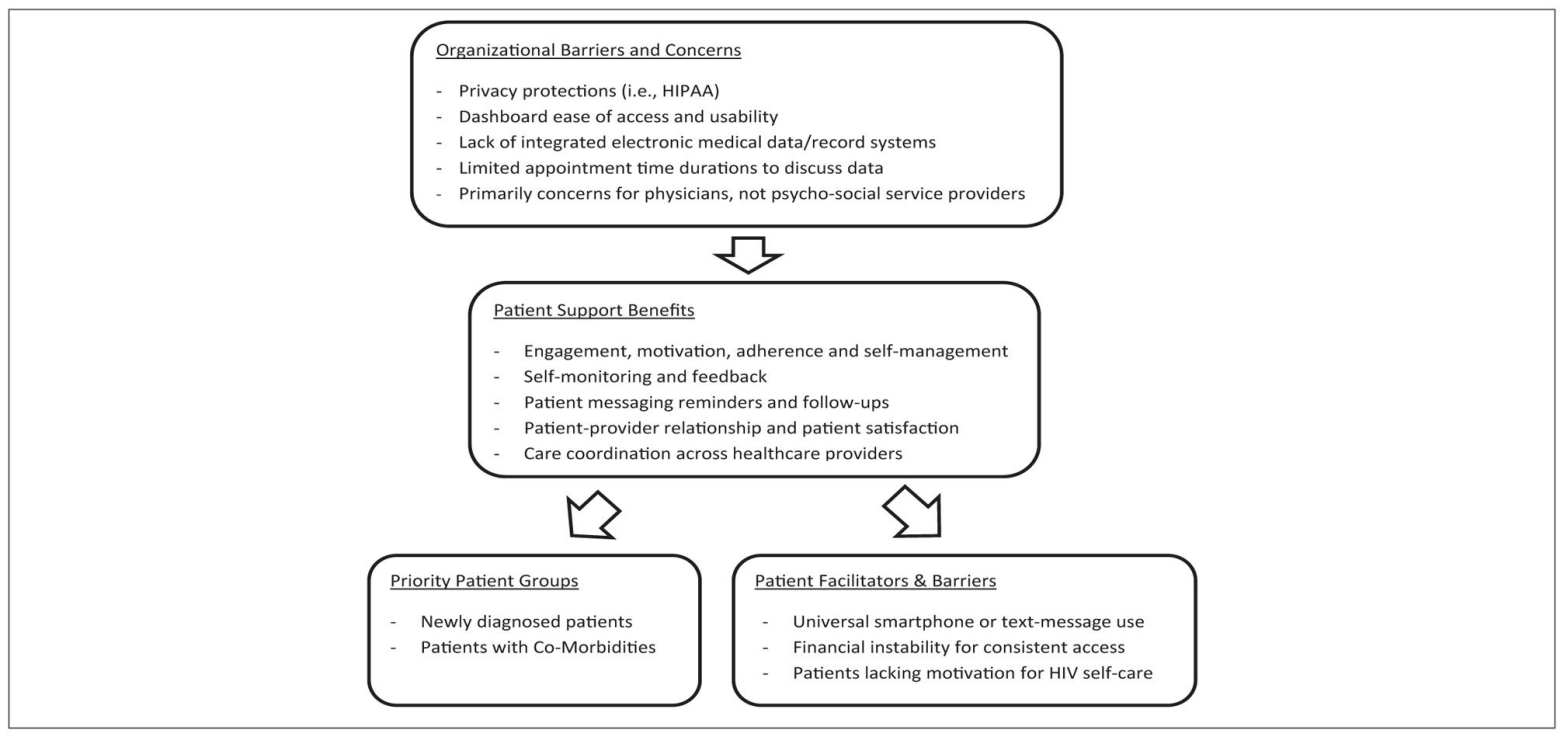
Main Themes around Motivators and Barriers on Mobile Phone Applications to Support Patient Self-Management and Web-Based Dashboards for Care Coordination

HCPs discussed themes regarding potential utility and value of mobile phone and web-based dashboard technology tools, with themes broadly focused on both patient-focused and provider-focused domains. Themes related to facilitators and barriers for adoption and use of technology tools were similarly clustered around factors specific to patients and providers, but also included privacy-related barriers for technology-based communications. The following themes were revealed during the in-depth interviews, as highlighted in figure 1.

### Patient support domains

#### Supporting patient engagement, motivation, and self-management through self-monitoring and feedback

HCPs thought that mobile technologies could be useful to enhance patient engagement, self-management, and satisfaction, as one physician noted:

“I think that, you know, if you’re able to....accomplish....developing the tool, that it will improve the patient’s engagement because if they engage with this, and then we look at it and we address it, then that will be validation for the patient that, ‘Oh, my input is important and they’re trying to do something about it.’ Whether or not you actually fix anything, you know, often times just listening to somebody is enough, so that you can communicate the sense of, you know, ‘We’re trying,’ you know?” (PPT04, MD).

One primary function of self-monitoring is to support behavior change through self-awareness and self-tracking, as one psychosocial service provider stated:

“Tracking is a valuable tool for people who are trying to make behavior change and see progress. You get some sort of immediate feedback and you get a visual feedback, and it helps with mindfulness and awareness.” (PPT02, EBI Coach).

HCPs also noted that enhancing patient opportunities for self-management through mobile self-monitoring tools could also enhance patient motivation, as one licensed vocational nurse noted:

“I would hope that in this day in HIV [care] that patients want to not have to come into the doctor all the time, and they’d want to be able to manage themselves and stay at home if they’re able to monitor, and take initiative of their own care. That’s something that they can feel good about themselves. I think they would be more apt and inspired to want to use something like that. I think patients, when they feel like they have to come into the office all the time, they do get discouraged.” (PPT10, LVN).

Feedback from providers based on mobile self-monitoring data was suggested as a primary opportunity to enhance patient engagement and motivation, as noted by one LVN:

“You know, the key thing is if you have a patient engaged in care, the way to keep them in care is giving them feedback, ‘You’re doin’ a good job.’ And so, yeah, I’ll give ‘em a call. Free will. I mean, that’s the nurses job, you know, you gotta keep your patients motivated.” (PPT10, LVN).

HCPs also noted that feedback could be transmitted by automated and electronic features, for example, by using data on patients’ trends in their progress, and with rewarding and motivating features, as one nurse case manage noted:

“…for my personal healthcare, I always have access to my labs online, and I think a resource like that, maybe either through the app where they could see their labs or access a website where they could see their labs and they could see a trend, and maybe give them a number of stars or a smiley face or something, I think would be tremendously helpful too. So that patients know that, you know, those taking their medication every day does have some sort of benefit, even if they maybe necessarily don’t feel better. But, just seeing that they’re trending really good and it gives them a score or a grade or something like that, I think would be awesome” (PPT12, NCM).

Overall, HCPs in different roles indicated the potential value of mobile self-monitoring and feedback, but they also suggested that simple reminder functions could also be useful.

#### Patient messaging for reminders & follow up

HCPs identified that the proposed application’s queries would also function simply as a reminder. For example,

“I think it’s something good to make someone aware when to take their medications, ‘Oh, I need to take…[medication],’ you know, a reminder.” (PPT05, MD)

Some HCPs also suggested enhancing simple reminder functions with personalized and automated messaging, such as for appointments, test results, problem solving, rewards and encouragements, and general follow-up. For example, one nurse case manager noted:

“I’ve always thought that one great thing would be for us to be able to send them text messages or reminders, especially for lab appointments, appointments with their therapist, appointments with their provider, or just any appointment in general. Even to remind them that they have a financial screening due. There are some patients who hardly ever talk on the phone but they’ll send a text message. So, you know, sometimes if you just need to say, “I’m just reaching out, following up, hope all is well.” I think if they [mHealth applications] are a way to link the application to a website or for us to send them, maybe, just a quick message…” (PPT12, NCM)

Overall, HIV HCPs recognized the potential value of mobile phone self-monitoring and feedback tools for patients, as well as for supporting provider-patient counseling.

#### Self-monitoring data and visual feedback to support provider-patient counseling

The results above highlight insights into the patient-facing aspects of mobile phone support tools. HCPs also recognized how tools could support provider understanding of their patients’ behavioral patterns. For example, one physician noted:

“It probably would be good for me as a tool to see what’s goin’ on like, ‘Why so many variations or variabilities as far as why are you taking it [medication] at this time and not the other time? You know, were you adherent to it?’” (PPT05, MD)

Psychosocial services providers, in particular, recognized the potential of using patient self-monitoring data in their counseling activities, as one psychosocial service provider noted:

“I could see it [mHealth] being a good therapeutic tool to be able to collect that data and have a visual that you could look at with the client in session.” (PPT02, EBI Coach)

A psychosocial case manager similarly noted:

“Yeah, I could see that […] using it [mHealth technology] as a tool to just reflect on, “What’s going on and how’s it going?” … they like that sort of thing and they enjoy havin’ that kind of interaction, I’d be happy to use that as a tool.” (PPT01, PCM)

In addition to psychosocial services providers’ roles and expertise related to counseling, their appointment durations also support opportunities to utilize patient monitoring data and visualization dashboards for counseling, as one psychosocial case manager noted:

“Visual aids really help to kind of illustrate what you’re trying to explain, or condense, or motivate a patient…we have 40 minutes [sessions]….I think you could set aside a good chunk of time to devote to reviewing that kind of information.” (PPT08, PCM)

Notably, although this PCM suggested time available to use mobile technology tools, physicians and nurse practitioners reported concerns about lack of time available to counsel patients, which are discussed below in relation to barriers and facilitators to use and adoption.

#### Care coordination

HCPs also suggested how patient mobile self-monitoring data could be used to support care coordination within their MCC teams, as one physician noted:

“If an app that the patient can interact act with on their own time that can help to understand that dynamic, and then it can be gotten into the hands of the medical team, like in this huddle, that says, ‘Oh, well….John has never been able to get his viral load under a thousand for the last year and, previously, he used to be really good, and look at the trend in this anxiety or stigma marker that he’s been monitoring for the last several’....well, maybe there’s somethin’ there.” (PPT04, MD)

HCPs also suggested that technology tools could facilitate coordination across other services and also to be able to follow up with patients as suggested by one nurse case manager below:

“You know, we work as a team. I would talk to the psychosocial care manager, we also have a person in our clinic who manages the crystal meth recovery services and the other substance abuse recovery system. So if we receive that [mHealth] data, we can always send them a message or an email and say, ‘Hey, you know, this patient that you’re monitoring, you know, stated via the mobile website that he was feeling despondent and depressed, and that he used crystal meth, and there might be a good chance, or this might be a good opportunity just to follow up with him and see how he’s doing and see what’s goin’ on to make sure that he’s okay.’” (PPT12, NCM)

### Priority patient groups and monitoring domains

#### Newly diagnosed patients

HCPs suggested that intensive self-monitoring would not be useful for all patients, but rather for patients with high self-management needs such as those newly diagnosed with HIV. For example, one psychosocial case manager noted:

“Yeah, I mean, especially with the ones who are newly diagnosed, that might be good, because there could be a lot goin’ on at the beginning and then as they’re startin’ to get a grasp of what it means for them, what it means with the friends or family they’re talking to or not talking to about, and that just hopefully getting a handle on what it means for them to be HIV positive, that, I see, especially with the newer, the newly diagnosed that that would be useful.” (PPT01, PCM)

Similarly, nurse case managers noted that most newly diagnosed and linked-to-care patients become self-managed (i.e., adherent, virally suppressed) within six-months to one year:

“If a patient has a moderate acuity, we have to reassess them in six months…Most of my patients that are moderate, initially, will come back self-managed…So, usually, I would say most of ‘em six months, sometimes a year.” (PPT12, NCM)

Thus, mobile phone support tools may be most useful to help newly diagnosed patients adapt and cope with the transition to managing their HIV infection as a chronic illness.

#### Patients with co-morbidities

HCPs also suggested that patients with co-morbidities would be a high priority for self-monitoring and self-management support with mobile technologies. For example, one nurse case manager noted:

“We have some patients that are co-infected with Hep C, so maybe tracking their Hep C viral load would be great. We have patients that are diabetic, so tracking their AIC or capillary blood glucose would be great. […]For patients that are hypertensive, maybe being able to track, you know, their most recent vital signs, or maybe even having a function where they can check their blood sugar or their blood pressure at home and putting it into the app, and then maybe it could communicate that information back to us. That would probably be great too.” (PPT12, NCM).

One physician expressed interest and support for advocating use of mobile technologies with patients with comorbidities but couched in concerns about patient motivations:

“I would be pushing it, if something like this were available, I would be pushing it with a bunch of patients…I just wonder whether my patients would even take the time to track stuff. I think most of them wouldn’t be very motivated to do that. But, if they could, it would be very useful in diabetes. They could do food diaries, blood sugar stuff, and medication compliance. They could do that on an app, and [if] they actually did it, it would probably give us some useful information. It would also be useful in people who, for instance, have insomnia, and you want to take a diary about their use of stimulants on a daily basis, their daytime napping, that kind of thing, just to put together a sleep hygiene pattern. People don’t usually remember that stuff, did I take a nap the day I had problems sleeping? So, that’s another scenario where it might be useful.” (PPT07, MD)

### Patient factors in facilitators and barriers domains

#### Mobile phone ownership

HCPs noted that the primary facilitator to mobile phone self-monitoring and self-management support for patients was perception of nearly universal mobile phone use by patients, as one licensed vocational nurse noted:

“If it’s something that maybe if it’s on their cell phone, they’re more apt to do it, you know? People are very electronic driven these days. So….I think it’s a text message generation and applications are like the way to go.” (PPT10, LVN)

Text messaging was also noted as a common and possibly preferred method for communication for some patients:

“I mean, youth these days, especially, it’s all about texting. Hardly anyone calls anybody anymore, you know? So, I could see how, for most people, that would be what they want, you know?” (PPT01, PCM)

Smartphone ownership was also noted as being very common, even among homeless patients, as one licensed vocational nurse noted:

“Yeah, I feel it would work well. Most people have nowadays, a smartphone, you know, and with the MCC program, ‘cause it’s a program that tries to help them with housing, and from what I understand…homeless patients, you think they, some of them don’t have cell phones, actually most of them do have cell phones anyway. They’re homeless but they have smartphones. So, I think, yeah, it definitely works well.” (PPT09, LVN)

#### Financial instability and lack of continuous phone access

Unstable finances were mentioned as a concern or barrier to mobile phone applications for self-management for some patients, as one EBI Coach noted:

“… also the instability of people being able to have a phone. I mean, there are a lot of our clients, who if they had the smartphone that would be able to have an app but they may not be able to hang on to it, or people’s phone bills are getting cut off and turned on pretty regularly. And so, it would require a certain…people who have financial stability…I think people do recognize the barriers that not having a phone creates for engaging in medical care. I mean, it’s really huge. We’ll have clients who, their phones are cut off - we have no way of getting in touch with them, you know?” (PPT02, EBI Coach)

#### Lack of patient motivation

A primary concern or potential barrier was the lack of motivation among some patients to engage in care and self-management, including the proposed mobile applications, as two physicians noted:

“I struggle with this issue a lot, of like the base motivation of folks. I get kind of discouraged and in despair about it a lot of times. I mean, I work a lot in prevention, so it’s like, what? People are still engaging in risk and all that stuff….I have to drag ‘em back into the clinic frequently. I mean, this isn’t everybody, but these are the ones that stick in your mind, you know. They expect another six months of refills without having any kind of lab, monitoring, or interaction with the doctor. And, we don’t think that’s close at all to the standard of care. And, it’s, you know, there’s a certain percentage of patients where, you know, I just have to babysit, kind of to pull them back in the right time and, I don’t know what percentage that is, say 25 percent. The percentage of patients who I think would be really willing and amenable to getting more involved through an app or something is, in my panel, is probably 10 [%].” (PPT 04, MD)

“I’m skeptical. But, yeah, it’s hard for me to read this younger generation, you know, ‘cause they do seem to be really willing, sometimes, to like do stuff that’s oriented towards their hand-held device. That mystifies me. (laughter)” (PPT07, MD)

### Organizational barriers for privacy protections in electronic communications domains

HCPs noted prior experiences that indicate patients’ desires to receive their health information electronically, such as test results by email, but that under current rules and systems providers can only provide such information in person or by mail, as a psychosocial case manager, licensed vocational nurse, and EBI Coach all noted:

“I try to avoid emails at all costs unless someone is really like this is how they prefer to be contacted because things are written down and it’s not - what’s the word for it? It’s not protected over email. You know, it’s not encrypted. It’s not encrypted. It’s not protected. Although, we just learned last Friday, a way that it can be encrypted. But in general, I’d rather just do the best I can for HIPAA compliance to like, to not have anything fall through the cracks in terms of that. So, only if they really want it. So, there’s only a couple people. There’s only like three or four people out of all the people I’ve seen who, here and there, there’s an email correspondence. Yeah, and I do not text. That’s not part of what we can do or are allowed to do.” (PPT01, PCM)

“I’ve only emailed patients general information. Sometimes they say like, you know, like, ‘You can go ahead and email me the doctor, specialist doctor’s name, phone number, and address.’ I can just do that. It’s not personal information. It’s just a doctor’s, specialist’s name and phone number. But, when, sometimes like I got (inaudible), sometimes we have patients saying like, ‘Oh, I just need my lab work, can you email it to me?’ I can’t do that because I don’t know exactly if the email, I might misspell one letter and it goes to the wrong email and then we get in trouble. (PPT09, LVN)

“We’ll mail them but that’s not, you know, an email’s not a secure way to communicate with clients, otherwise, we might do it more. We do email some clients if we get their consent.” (PPT02, EBI Coach)

#### Data dashboard ease of access

Another major concern was ease of access to using the web-based dashboards and reporting, which would be critical to making them useful in health care settings, as one physician and one nurse practitioner noted:

“I think maybe part of the only thing, for me, is it would simplify my life a lot if I were to have this data but it were presented very easily for me, just to go ahead [and] access [it], not to have to figure out, ‘Okay, well do I have to fill it out myself?’ As long as it will be self-populated and I wouldn’t have to necessarily do anything extra.” (PPT05, MD)

“Depending on how much time it would take to review it, you know what I mean? If it was quick and easy - absolutely. But, if it took me having to, oh my God, I’ve got to log in here, log in there, blah-blah-blah-blah-blah-blah, and then, you know?” (PPT06, NP)

#### Lack of integrated electronic systems

Of the twelve HCPs interviewed eight (3 MDs, 1 NP, 1 LVN, 1 LCSW, 2 PCMs) talked about feeling overwhelmed by existing electronic medical record (EMR) systems, particularly the poor integration of multiple electronic systems.

“So, we have this EMR, right? But nothing is centralized on that EMR. So, if I have a disability for a patient, I have to go to the disability site, right? It takes forever. Then, I gotta do an e-consult, so I have to check in on e-consult, then I have to go to all these external places. So, if it was one centralized thing, that would be different, but it’s not. So, you have to go out of this program and go into that program[…] and they don’t talk and they don’t communicate, which like our EMR doesn’t communicate with our pharmacy, so it’s like you can’t do one on the same screen, you have to come out of one to go into the other, and so that’s where it becomes really cumbersome…So, then it becomes burnout issues, right, you know? Oh my God, how am I, I don’t have time to do that.” (PPT06, NP)

#### Intersection of burdens from electronic systems and limited appointment times

All of the MDs and the NP also mentioned barriers related to limited time with patients under current reimbursement structures (i.e., 15–20 minute appointments) to counsel patients, and that complicated web-based dashboards would further exacerbate the time concerns:

“Well, stuff is being laid on just endlessly. And, I don’t know why doctors are puttin’ up with this because the length of time, their appointment times are also shrinking, you can’t, it’s like a no win situation. And, so I said, ‘I’m not takin’ anything more on my plate until we get the operational problems fixed.” (PPT 05, MD)

“So, you know, I think that medical providers, in general, in this day and age of electronic medical records are increasingly frustrated with all of the electronic interface problems to document the medical encounter. And, I know from having read comments from various websites that the problems and complaints are not restricted to just our EMR in this institution. I think it’s across the board, most people who work with electronic medical records have significant levels of frustration. So, what that leads to is a, kind of a constant level of anxiety among the providers as to getting through a medical visit where the provider’s list of things that they want to accomplish during that visit is reasonably accomplished. The patient’s list of items that the patient wants to accomplish is accomplished. And you then have to document things that our funders require. And, all of those tensions and pressures have to be met within a limited period of time, like 20 minutes. That’s a pretty tall order. So, whenever somebody comes and says, ‘I’ve got a new way of helping you get information that’s important to the patient,’ the first thing that’s gonna go down in front of the provider’s eyes is a curtain that says, ‘Oh, great! More stuff that I got to deal with in a limited time.’ However, if there can be a tool that doesn’t require the provider to navigate through various different screens…but that transmits useful information, of course they’re gonna be interested in it.” (PPT04, MD)

Despite these concerns, HCPs interviewed expressed interest in trying mobile technology tools, and in their care teams, with high-need sub-groups of patients. Again, interests tended to vary by HCPs’ role. The two NCMs (RNs) and two LVNs expressed the most interest in symptom and adherence monitoring, consistent with the scope of their roles on the MCC teams. Behavioral intervention providers (PCMs, EBI Coach) expressed the most interest in using monitoring and dashboard tools, which is likely enabled by 40-minute visit times and their role responsibilities.

## Discussion

The results of this study indicates that opportunities for integrating patient mobile monitoring and provider dashboard technologies in patient-centered medical homes, and specifically for HIV care, are feasible and acceptable to HCPs and particularly for patients newly initiating treatment or with co-morbidities. There were notable concerns around patient motivation and technology access, and for provider time burdens and adding another electronic system to workflows. Despite concerns, HCPs were open to trying the technologies if the burdens were allocated acceptably. Specifically, psychosocial or behavioral health services providers were most open to using patient mobile monitoring technologies and would likely drive technology adoption by having primary responsibilities for supporting behavior change and 40-minute visit times with patients typically. Nurses express some support to drive technology adoption, particularly when applied to medication adherence, symptoms, and general health-related behaviors (e.g., diet, sleep). These results complement results from similar prior studies with other chronic conditions that identify technology barriers such as system readiness and liability concerns, but still finding support from many HCPs [8,10].

There are several limitations in this study. First, the sample size is small, and so may not have captured the full range of perceived benefits and barriers and qualitative studies in general are not designed to make inferences on the frequency or commonality of themes identified. Inferences suggested by variations in themes by HCP roles are consistent with their roles and expertise, but should be interpreted with caution. Furthermore, since participants were volunteers and represented about half of the HCPs on the care teams, except for MD/NP team leaders, self-selection biases likely result in emphasis of more motivated and optimistic HCPs who might be considered potential early adopters rather than fully representative of their peers. Another limitation is that this preliminary feedback is based on hypothetical discussion of proposed tools (used in prior pilot studies with HIV-positive persons) rather than the HCPs’ actual user experiences. Actual user-experiences are required to make reliable inferences on acceptability, efficacy and potential for scalability.

## Conclusion

Overall, HCPs interviewed expressed interest in trying mobile technology tools with high-need sub-groups of patients, such as moderate- to high-acuity, newly diagnosed, persistently non-adherent, and dually diagnosed patients, or those receiving behavioral interventions (e.g., for mental health; alcohol, tobacco or drug use; sexual risk behaviors). Mobile phone self-monitoring tools have the potential to support motivation, behaviors, and self-management for people living with HIV, and potentially, to enhance and accelerate treatment and intervention outcomes through integration into treatment settings and HCPs service delivery and care coordination [18,19]. The results of this study indicate that there is sufficient interest by HCPs in HIV treatment and care programs to further develop and test mobile phone monitoring, visualization dashboards, and patient messaging tools to enhance patient self-management, and care delivery and coordination.
